# Epoxide-Based Synthetic Approaches toward Polypropionates and Related Bioactive Natural Products

**DOI:** 10.3390/ijms24076195

**Published:** 2023-03-24

**Authors:** Raúl R. Rodríguez-Berríos, Stephen R. Isbel, Alejandro Bugarin

**Affiliations:** 1 Department of Chemistry, University of Puerto Rico, Rio Piedras Campus, P.O. Box 23346, San Juan 00931-3346, Puerto Rico; raulr.rodriguez@upr.edu; 2 Department of Chemistry & Physics, Florida Gulf Coast University, 10501 FGCU Boulevard South, Fort Myers, FL 33965, USA

**Keywords:** epoxide, polypropionate, antimicrobial, natural products, total synthesis

## Abstract

Polypropionate units are a common structural feature of many of the natural products in polyketides, some of which have shown a broad range of antimicrobial and therapeutic potential. Polypropionates are composed of a carbon skeleton with alternating methyl and hydroxy groups with a specific configuration. Different approaches have been developed for the synthesis of polypropionates and herein we include, for the first time, all of the epoxide-based methodologies that have been reported over the years by several research groups such as Kishi, Katsuki, Marashall, Miyashita, Prieto, Sarabia, Jung, McDonald, etc. Several syntheses of polypropionate fragments and natural products that employed epoxides as key intermediates have been described and summarized in this review. These synthetic approaches involve enatio- and diastereoselective synthesis of epoxides (epoxy-alcohols, epoxy-amides, and epoxy-esters) and their regioselective cleavage with carbon and/or hydride nucleophiles. In addition, we included a description of the isolation and biological activities of the polypropionates and related natural products that have been synthetized using epoxide-based approaches. In conclusion, the epoxide-based methodologies are a non-aldol alternative approach for the construction of polypropionate.

## 1. Introduction

**Figure 1:**
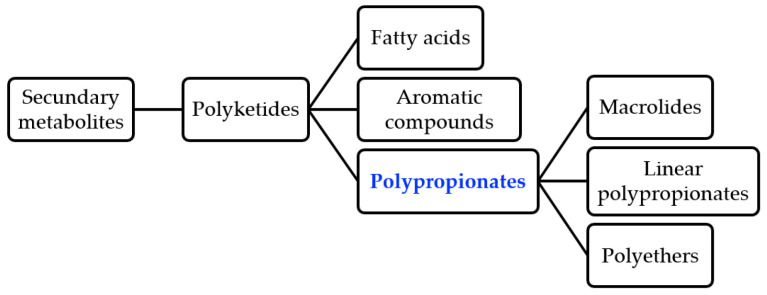
Types of polyketides in natural products.

All living organisms have the capacity to transform and interconvert a variety of molecules using two types of metabolic pathways, primary and secondary metabolism, with this review focusing primarily on the latter. In general, the majority of organisms produce different kinds of secondary metabolites in a limited distribution and play an important role in the well-being of the producer [1]. Polyketides are secondary metabolites derived from the acetate pathway and are an important large family of natural products biosynthesized by animals, bacteria, fungi, and plants. This process occurs through the Claisen condensation of C_3_-units catalyzed by the enzyme polyketide synthases (PKSs) [2]. Polyketides include groups of fatty acids, aromatic compounds (e.g., anthraquinones, tetracyclines), and polypropionates (Figure 1). Polypropionates are further classified into subgroups including macrolide antibiotics, linear polyketides, and polyethers [3]. In fact, more than 10,000 polyketides have been reported and about 1% of them possess drug activity (typically antibiotic, anticancer, antifungal, antiparasitic, immunomodulatory action, and cholesterol lowering agents), which is approximately five times more bioactive than the average of other natural product families [3,4]. Around 20% of the top-selling, small-molecule drugs are polyketides [5].

**Figure 2:**
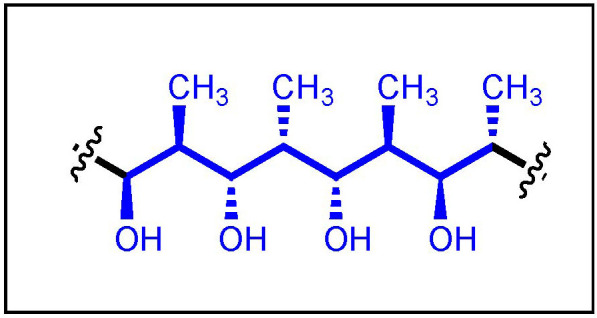
General polypropionate structure (i.e., stereooctad).

The propionate unit consists of an aliphatic chain with alternating methyl and hydroxy groups with a specific configuration at each carbon (Figure 2). For decades, the construction of polypropionate chains has attracted the great interest of synthetic organic chemists due to the challenge represented by the elaboration of their array of contiguous asymmetric centers. As the number of stereocenters increases, the difficulty of synthesizing these compounds also increases. In 1987, the term “stereo-*n*-ad” was introduced by Hoffman, in which *n* refers to the number of asymmetric carbons within the propionate chain [6]. For example, three and five chiral centers represent a stereotriad and stereopentad, respectively.

**Figure 3:**
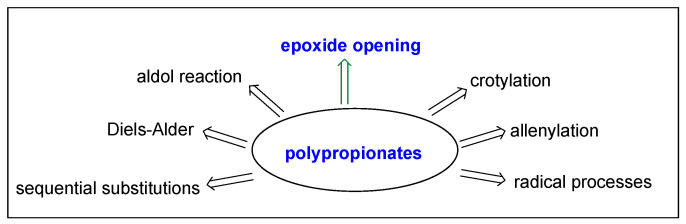
Methods for the stereoselective synthesis of polypropionates.

Aldol type reactions represent one of the primarily used methods for the regio-, stereo-, and enantioselective formation of propionates that has been developed [7,8,9]. Furthermore, other methods for the stereoselective assembly of long sequences of stereocenters in polypropionates have been developed. These methods include crotylations [10,11,12], allenylations [13,14,15], selective radical processes [16,17,18,19], sequential substitutions [20,21], epoxide ring openings [22,23,24,25], Diels–Alder [26], and others [27,28,29,30] (Figure 3).

In this review, we describe the structures, classification, and antimicrobial activities of approximately thirty polypropionate natural products that have been synthesized using epoxide-based approaches by different research groups from 1980 to 2020. Some research groups reported their epoxide-based methodologies for a partial and/or total synthesis of polypropionates. Therefore, this review describes and depicts the general epoxide-based approach of each group including key steps to construct the polypropionate units, an example in the synthesis of a polypropionate fragment, and all the polypropionate fragments and/or natural products reported by each group.

### 1.1. Isolation, Structural Characterization, and Antimicrobial Activities of Polypropionates

#### 1.1.1. Macrolides

**Figure 4:**
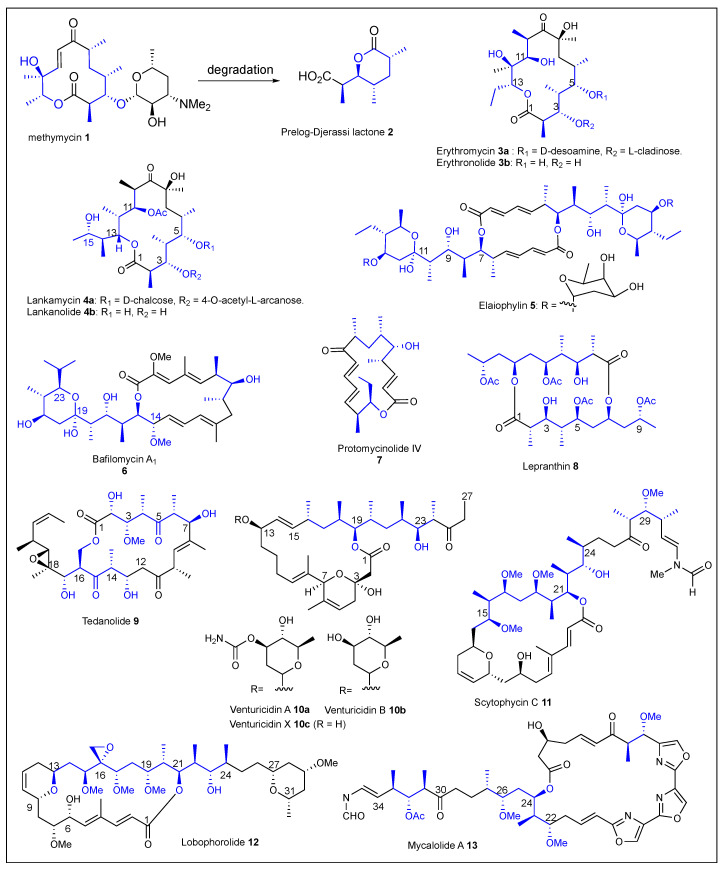
Structures of reported macrolides **1**–**13**.

Macrolides are a large family of polypropionates in which the majority exhibit antibiotic activity, and their structure is characterized by a range of 12-, 14-, 16-, 18-, or 22-membered macrocyclic lactone rings. For example, methymycin **1** (Figure 4) is a 12-membered macrolide antibiotic biosynthesized by *Streptomyces venezuelae* [31]. Prelog–Djerassi **2** (Figure 4) is a 6-membered polypropionate lactone that was obtained as a degradation product of **1** and retains the configuration of the four chiral centers corresponding to the C1-C7 polypropionate fragment. Erythromycin A **3a** (Figure 4) is a 14-membered macrolide antibiotic produced by *Saccharolyspora erythraea* and was isolated in 1952 [1]. Erythromycins show activity against Gram-positive bacteria, penicillin resistant *Staphilococus* strains, and are used to treat infections of *Legionella pneumophilia.* The mechanism of action consists in the inhibition of the protein biosynthesis in the organism. The structure was elucidated by spectroscopic methods and confirmed by X-ray crystallography [32]. Erythromycin A **3a** attaches at the C3 and C5 two sugars, d-desoamine and l-cladinose, respectively, and the aglycone structure. It is worth noting that erythronolide A **3b** does not have the corresponding glycoside linkages.

Lankamycin **5a** (Figure 4) was collected from a soil sample and isolated from various *Streptomyces* species: first in 1960 by Gäumann et al. from *Streptomyces* v*iolaceoniger* [33] and later in 1969 by Namiki et al. from the Kuji District of Ibari Prefecture, Japan from *S. spinichromogenes* var. *kujimyceticus* [34]. In addition, Kinashi et al. reported that *Streptomyces rochei* 7434AN4 produces lankamycin **5a** and other structurally unrelated polyketide antibiotics [35]. Lankamycin **5a** shows moderate inhibitory activities against *Mycobacteria* [34] and Gram-positive bacteria such as *Staphylococcus aureus*, *Bacillus subtilis*, and *Micrococcus luteus* [36,37]. The relative structure of the neutral 14-membered macrolide antibiotic lankamycin **4a** was determined by Keller et al. [38]. The molecule consists of a lactone ring, 11-acetyllankolide, which contains a polypropionate fragment with twelve stereogenic centers. The structure of lankamycin **4a** has two sugar moieties, in which d-chalcose is bound at the C5 and the 4-O-acetyl-l-arcanose is bound at C3 [39]. Lankanolide **4b** (Figure 4) is the aglycon of **4a**. Elaiophylin **5** (Figure 4) is a 16-membered macrodiolide isolated from different cultures of *Streptomyces malonosporus* and belongs to a group of C2-symmetrical macrodiolides [40]. The elaiophylin **5** structure was determined by extensive chemical degradation and nuclear magnetic resonance (NMR) studies, while X-ray crystallographic analysis revealed the relative and absolute stereochemistry [41]. The structure of **5** consists of a macrolactone, an intramolecular hemiacetal, and a polypropionate fragment with nine asymmetric carbons. Macrolide **5** exhibited potent antimicrobial activity against several strains of Gram-positive bacteria including *Bacillus subtilis*, *Staphylococcus aureus*, and *Corynebacterium diphtheriae*. It also revealed anthelmintic activity against *Trichonomonas vaginalis* [42,43].

Bafilomycin A_1_ **6** (Figure 4) was isolated in 1983 by Wagner from a broth of *Streptomyces griseus* [44]. Its relative stereochemistry was determined by Corey and Ponder in 1984 using extensive NMR analysis, and confirmed in 1987 using X-ray crystallography [45,46]. The structure of **6** represents a 16-membered macrolide with a tetraene core, a cyclic hemiacetal, a C14 methoxy, and a C23-isopropyl group within its polypropionate chain. Bafilomycin A_1_ **6** exhibited potent antifungal and antibacterial activity and is also the first known inhibitor of Vacuolar H^+^-ATPase (VATPase) [47]. Protomycinolide IV (**7**) (Figure 4) is the aglycone of Mycinamicins [48]. It is a 16-membered macrolide isolated from *Micromonospora griseorubida sp*. nov. that possesses excellent activity against Gram-positive bacteria [49]. The protomycinolide IV (**7**) structure is characterized by a short polypropionate chain that contains four chiral carbons of the six in total, a conjugate ketone, and macrocyclic lactone.

The macrocyclic bislactone, lepranthin **8** (Figure 4), was isolated from a species of crustaceous lichen *Arthonia impolita* (Ehrh.) Borrer by Zopf, approximately 119 years ago (in 1904). The structure was elucidated using NMR and X-ray crystallographic analysis in 1995 by Huneker et al. [50]. Lepranthin **8** consists of a 16-membered homo-macrodiolide and the polypropionate chain contains 12 stereogenic centers in which there are four secondary acetates. Biological activity studies for Lepranthin **8** have not been reported in the literature. Tedanolide **9** (Figure 4) is a very potent cytotoxic macrolide that was isolated from *Tedania ignis*, a very well-known Caribbean marine sponge [51,52]. The 18-membered macrolactone **9** was elucidated using high resolution fast atom bombardment/mass spectrometry (FAB/MS), infrared (IR), NMR spectroscopy, and X-ray diffraction. Tedanolide **9** exhibited good cytotoxicity against the KB cell line (ED_50_ = 2.5 × 10^−4^ ng/mL) and PS cell line (ED_50_ = 1.6 × 10^−5^ ng/mL) [51]. Furthermore, it has demonstrated strong antitumoral activities since it has been found to increase the life span of mice implanted with lymphocytic leukemia by 23% [53]. Venturicidins A (**10a**) and B (**10b**) (Figure 4) are 20-membered macrolide antibiotics that were isolated from *Streptomyees aureofaciens*, exhibit a potent antifungal activity [54], and are specific inhibitors of mitochondrial ATPase [55]. The structures of venturicindins **10** were characterized by NMR, MS, and X-ray analysis. Specifically, venturicidin X (**10c**) (Figure 4) is the aglycone, and the R group of venturicidin A **10a** and B **10b** are 3-O-carbamyl-2-deoxy-D-rhamnoside and 2-deoxy-D-rhamnoside, respectively. These macrolactones contain a tetrahydropyran ring, ten asymmetric carbons in total, in which seven chiral carbons belong to the polypropionate chain.

Scytophycin C **11** (Figure 4) is a 22-membered macrolide isolated in 1986 by Moore et al. from the terrestrial blue-green alga *Scytonema pseudohofmanni* [56]. The structure of **11** contains 15 stereogenic centers, a dihydropyran ring bearing two *trans*-substituted side chains, a polypropionate chain, and a terminal *N*-methyl-*N*-vinylformamide moiety. Scytophycin C **11** exhibited potent activity against a variety of human carcinoma cell lines including solid tumors [57].

Lobophorolide **12** (Figure 4) was isolated from the Caribbean brown algae *Lobophora variegata* by Kubanek et al. in 2003 [58]. They characterized the lobophorolide **12** structure using IR, NMR, and MS analysis, which consists of a 22-membered macrolactone that has attached an aliphatic side chain with a tetrahydropyran ring-termini. Lobophorolide **12** displayed antifungal activity against *D. salina* and *L. thalassiae* as well as a high cytotoxicity toward the colon tumor cell line HCT-116 (IC_50_ = 0.03 μg/mL) [59].

**Figure 5:**
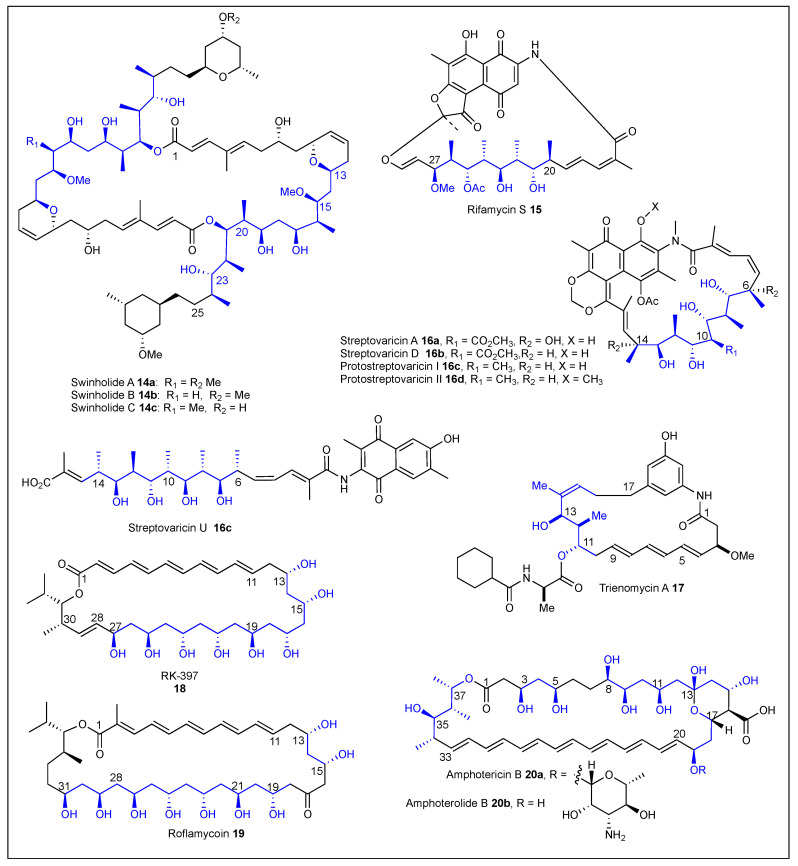
Structures of the reported macrolides **14**–**20**.

The isolation of mycalolides was reported in 1989 by Fusetani et al. from a sponge of the genus *Mycale* [60]. Mycalolide A **13** (Figure 4) has a 25-membered macrolide ring that incorporates the tris-oxazole, attached to an aliphatic polyketide side chain that contains eight of the eleven stereogenic centers present in this compound [61]. The relative and absolute configuration were determined through a combination of chemical degradation, extensive NMR analysis, and correlation experiments [62]. Mycalolide A **13** exhibits potent antifungal activity against a wide array of pathogenic fungi and cytotoxicity toward B-16 melanoma cells (IC_50_ = 0.5–1.0 ng/mL). It was also shown that it selectively inhibits the actomyosin Mg^2+^-ATPase, acting as an acting-depolymerizing agent [63]. Swinholides A–C (**14a-c**) (Figure 5) was isolated from the marine sponge *Theonella swinhoei* [64]. The structure consists of a 44-membered dimeric macrolide, with four tetrahydropyran rings, two conjugated double bonds connected to each ester, and the polypropionate chains had nine chiral centers [65]. Swinholides **14a-c** exhibit high cytotoxic activity against a variety of tumor cells and possess antifungal activities [50].

Another group of macrolides named ansa lactams, possess an aromatic ring (naphthalene, naphthoquinone, or benzene) connected to the aliphatic chain in non-adjacent positions. Rifamycin S **15** (Figure 5) is a member of the lactam ansa macrolide antibiotic that was isolated from *Norcodia mediterranei* in 1951 [66,67]. This macrolactam **15** consists of twenty-four-member rings that contain a naphthoquinone portion that is connected by an aliphatic polypropionate chain with eight consecutive stereogenic centers. Rifamycins have shown activity against Gram-positive and Gram-negative bacteria with the most valuable activity toward *Mycobacterium tuberculosis*, leprosy, and meningitis. Rifamycin activity works through the inhibition of the DNA-dependent RNA polymerase and at high concentration through the inhibition of the RNA-dependent DNA polymerase of retroviruses [1,68].

The streptovaricin anasamycin antibiotics A–K were first isolated from *Streptomyces spectabilis* in 1957 by Siminoff [69]. Streptovaricins **16** (Figure 5) showed activity against Gram-positive, Gram-negative bacteria, and *Mycobacterium tuberculosis* [70]. Ten years later, Rinehart et al. was able to characterize the macrolactams by NMR, chemical derivatization studies, and X-ray analysis [71]. The structures of streptovaricin A **16a**, D **16b**, and U **16e** (Figure 5) consist of a napthoquinoid core and a polypropionate chain with nine contiguous stereogenic centers, five of which have a challenging all-*anti* relative configuration [72]. Interestingly, the streptovaricin U **16e** ansa segment replaced the C10 carbomethoxy for a methyl group and streptovarcin A **16a** has two tertiary alcohols at the C6 and C14 positions. Streptovaricin D **16b** inhibits the RNA-directed DNA polymerase and was the most active against reverse transcriptase (0.25 μmol/mL for 70% inhibition) in comparison to all of the streptovaricins [73]. Streptovaricin U **16e** is a unique, open-chain streptovaricin that inhibits Rauscher leukemia virus RNA-dependent DNA polymerase (40% inhibition at 200 μg/mL) [74]. Protostreptovaricins I–V were isolated after various chromatographic purifications of fractions from the streptovarin complex [75]. The protostreptovaricins are precursors of streptovaricins that are active inhibitors of reverse transcriptase. The ansa chain of **16e** and protostreptovaricin I **16c** and II **16d** (Figure 5) were the same and only differed in the substitution of the napthoquinoid core. In fact, streptovaricin U **16e** is most likely the result of the enzymatic hydrolysis of protostreptovaricin I **16c** by *S. spectabilis.*

The ansamycin antibiotic trienomycin A **17** (Figure 5) was isolated and purified by Komiyama et al. in 1985 from the culture broth of *Streptomyces sp.* No. 83-16. Komiyama reported the physico-chemical characteristics, molecular formula, and biological activities against HeLa S_3_ (IC_50_ = 0.1 μg/mL) and PLC hepatoma (IC_50_ = 0.01 μg/mL) cells in vitro [76]. Then, the structures of trienomycins A **17**, B, and C were determined based on their spectroscopical and chemical properties [77,78]. Trienomycins are unique ansamycin lactams with a stereotriad polypropionate segment, a triene, an 1,3,5-trisubstituted benzene, and *N*-hexahydrobenzoylalanine moieties in the structure. Trienomycins do not exhibit antimicrobial activity against bacteria, fungi, or yeasts. In 1995, Otake et al. isolated trienomycins A **17**, B, and C *Streptomyces rishiriensis* T-23. They also characterized the structure of this mycotrienin antibiotic by NMR studies and reported cytotoxicity against L-5178Y (IC_50_ = 0.11 μg/mL) cells in vitro [79].

#### 1.1.2. Polyene Macrolides

Polyene macrolides are a larger group of macrolides characterized by the presence of a series of conjugated *E* double bonds and the macrolactone is in the range of 26–38 atoms. Polyenes have antifungal properties, and the medicinally important ones include the heptaene amphotericin B **20** (Figure 5) and the tetraene nystatin. In 1993, Osada et al. isolated a 32-membered macrolide, RK-397 (**18**), from a soil sample collected in Japan and produced by *Streptomyces* sp. 87-397 [80]. They reported the physico-chemical properties, NMR spectra, molecular formula, and biological activities of the oxo pentaene **18** [81]. The polyene **18** (Figure 5) showed cytotoxicity against tumor cells lines K-562 and HL-60, inhibiting human leukemia, and was active against filamentous fungi, yeast, and bacteria. Macrolide structure **18** consists of a conjugated pentaene and a large segment of 1,3-polyols. Roflamycoin **19** (formerly named flavomycoin) (Figure 5) is a 36-membered polyene macrolide that was isolated from *Streptomyces roseoflavus* and has antifungal activity [82]. Schlegel et al. determined the structure using spectroscopic and chemical degradation methods [83], and then reported the absolute configuration using the 13C acetonide method and synthetic correlation [84]. The structure consists of a conjugated pentaene, a ketone at C17, and a long 1,3-diol chain. Roflamycoin **19** is an ion channel-forming antibiotic that increases the membrane permeability only in the case of sterol-containing membranes. The channels are potential-dependent, have a short lifetime, and high conductance [85]. Amphotericin B **20a** (Figure 5) is a polyene antifungal antibiotic that consists of a 38-membered macrolide that was isolated from *Streptomyces nodosus* from the Orinoco River in Tembladora, Venezuela [86]. Amphotericin **20a** is active against yeasts and most fungi including *Cryptococcus neoformans, Candida albicans, Sporotrichum, Blastomyces dermatitidis, Histoplasma capsulatum, Coccidioides immitis*, and *Aspergillus fumigatus*. In addition, **20a** is used for the treatment of cryptococcosis, histoplasmosis, disseminated candidiasis, coccidioidomycosis, North American blastomycosis, aspergillosis, and sporotrichosis [87]. The structure of **20a** was elucidated in 1970 employing chemical manipulations and MS studies [88]. The absolute configuration of **20a** was established using X-ray single-crystal analysis [89]. The structure of macrolactone **20a** consists of a steretetrad polypropionate portion, 1,3-polyol segments, a cyclic hemiacetal substituted with a carboxylic acid, and a conjugated heptaene. Amphoterolide B **20b** (Figure 5) is the aglycone of Amphotericin B **20a**.

### 1.2. Linear Polypropionates

**Figure 6:**
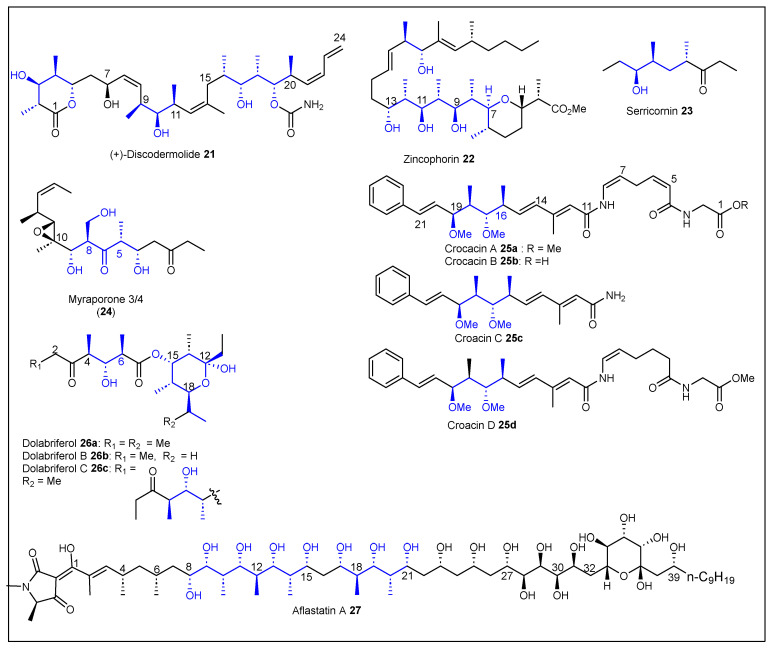
Structures of the reported linear polypropionates **21**–**27**.

Macrolide formation does not always occur during biosynthesis in some organisms such as marine mollusks, microorganisms, terrestrial plants, and insects, and produce a rare class of polyketide metabolites that can be classified as linear polypropionates **[90]**. (+)-Discodermolide **21** (Figure 6) is a linear polypropionate that contains a six-membered lactone that was isolated from the Caribbean marine sponge *Discodermia dissolute* [91]. This natural product has been approved for clinical trials regarding antitumoral activity and has also shown strong antimitotic, antifungal, and immunosuppressant activity [92]. The structure of **21** contains an amide and a polypropionate segment with 13 chiral centers. The absolute configuration was determined by analyzing the NMR data and X-ray results in 1990 [93]. Discodermolide **21** exhibited strong cytotoxicity against murine P388 leukemia and human lung adenocarcinoma A549 cell lines [90]. Zincophorin **22** (Figure 6) was isolated from a strain of *Streptomyces griseus* and the structure consists of a linear polyketide that contains a tetrahydropyran ring and an methylester termini [94]. This zinc-binding antibiotic (ionophore) possesses a remarkable in vitro activity against Gram-positive bacteria as well as against *Clostridium coelchii* and influenza WSN/virus [95]. (−)-Serricornin (4,6- dimethyl-7-hydroxy-nonan-3-one) **23** (Figure 6) is a sex pheromone of a female cigarette beetle (*Lasiodema serricorw* F.) that has been isolated and structurally characterized by chemical and spectroscopic evidence by Chuman et al. in 1979 [96]. Myriaporones (Figure 6) have been isolated from the western Mediterranean Sea marine organism bryozoan *Myriapora truncata* [97]. The NMR, IR, and FAB/MS data analysis were employed to determine the structures and stereochemistry of each myriaporone (1, 2, 3/4). Interestingly, myriaporones 3/4 (**24**) (Figure 6) were isolated as inseparable equilibrium mixture of acyclic and cyclic isomers, respectively. In 2004, the research group of Echavarren reported the total synthesis of all myriaporones and the relative and absolute configurations of these cytotoxic compounds [98]. Myriaporones 3/4 (**24**) were the most active and showed 88% inhibition of L1210 murine leukemia cells at 0.2 μg/mL [97]. Crocacins A–D (**25a-d**) (Figure 6) are unusual secondary metabolites in which the biosynthesis of the structure consists of a mixture of polypropionate and peptide building blocks [99]. They are potent antifungal and cytotoxic compounds isolated from myxobacteria *Chondromyces crocatus* and the bacteria *Chrondomyces pediculatusi* by Reichenbach et al., which named the structures of **25** as (*Z*)-enamides [100]. The structure of crocacins **25a-d** has a common polypropionate steretetrad fragment (C16–C19) and the conjugated (*E*,*E*)-dienamide motif (C11–C15). The relative configuration of crocacins **25a-d** was confirmed by Jansen et al. in 1999 [101]. Their absolute configuration was later confirmed by its first total synthesis in 2000 by Rizzacasa et al. [102]. Only crocacins A **25a** and D **25a** have shown high biological inhibition of the electron transport chain in a beef heart mitochondrial respiration assay. They also inhibited the growth of several yeast and fungi in vitro such as *Plasmopara viticola* and *Phytophthora infestans* [103]. Dolabriferols **26a-c** (Figure 6) were isolated by Gavagnin et al. from the anaspidean mollusk *Dolabrifera dolabrifera* [104]. The structure of dolabriferol was determined by spectral methods and the relative stereochemistry was determined by X-ray analysis and consisted of two polypropionate subunits linked by an ester. Similar compounds, dolabriferol B **26b** and C **26c**, were isolated by Rodriguez et al. from a Caribbean mollusk *Dolabrifera dolabrifera* from Puerto Rico and characterized using spectral data and X-ray analysis [105]. Dolabriferol, in general, did not display any significant activity with the exception of Dolabriferol B **26b** and **26c** at a concentration of 128 μg/mL, which showed an inhibitory activity (39% and 93%, respectively) against *Mycobacterium tuberculosis* H37Rv. Sakuda et al., in 1996, isolated aflastatin A **27** (Figure 6) from *Streptomyces sp.* MRI142, which is an inhibitor of aflatoxin biosynthesis, but does not inhibit the growth of *Aspergillus parasiticus* [106]. First, they reported the preliminary structure of **27** after employing chemical degradation and spectroscopic analysis, and later confirmed and revised the absolute configuration of all 29 chiral centers [107,108]. The tetramic acid derivative **27** consists of a long and linear polyol and polypropionate chain with a tetrahydropyran ring moiety.

#### 1.2.1. Polyethers

**Figure 7:**
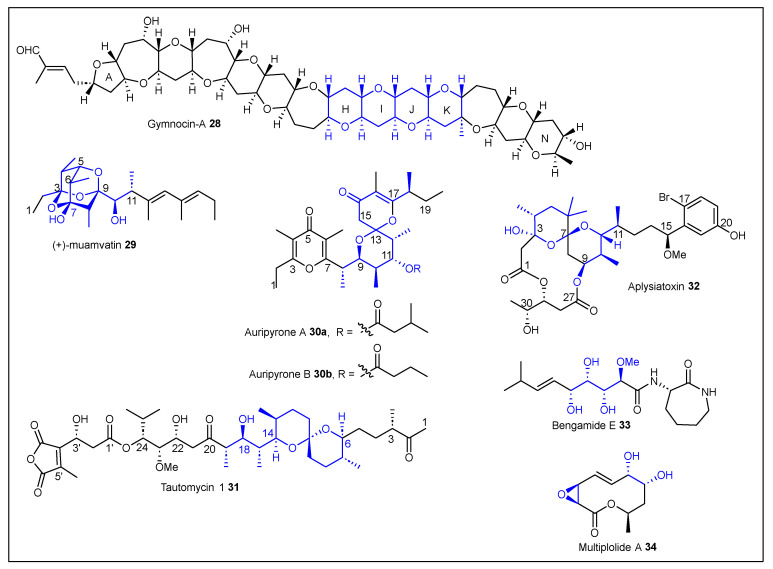
Structures of the reported polypropionates **28**–**34**.

Polyether antibiotics are a large group of secondary metabolites that show a broad spectrum of bioactivities such as antibacterial, antifungal, antiparasitic, antiviral, and tumor cell cytotoxicity [109]. These are characterized by the presence of several tetrahydrofuran, tetrahydropyran, and oxepane all-*trans* fused rings. Gymnocin-A **28** (Figure 7) is classified as a polycyclic ether and was isolated from the red tide dinoflagellate, *Gymnodinium mikimotoi*, at Kushimoto Bay, Japan in 2002 [110]. The structure of gymnocin-A **28** was determined by NMR and FAB/MS spectrometry and consists of a linear array of 14 fused ether rings and a 2-methyl-2-butenal side-chain. The absolute configuration was assigned via Mosher ester analysis. Gymnocin-A **28** showed potent cytotoxicity (IC_50_ = 1.3 μg/mL) against mouse leukemia P388 cells [111]. Muamvatin **29** (Figure 7) is a marine polypropionate that contains a unique 2,4,6-trioxaadamantane ring skeleton that was isolated from the Fijian pulmonated mollusc *Siphonaria normalis* by Ireland et al. in 1986 [112]. They elucidated the structure by employing NMR analysis, specifically, the relative stereochemistry of the trioxaadamantane ring (tricyclic ketal) constituents was determined based on the results of a two-dimensional NOE experiment. The absolute configuration of (+)-muamvatin **29** was determined by chemical degradation studies [113] and after the total synthesis of **29** reported by Paterson and Perkins in 1993 [114]. Yamada et al. reported the isolation of two new polypropionates, auripyrones A **30a** and B **30b** (Figure 7), from the sea hare *Dolabella auricularia* (Aplysiidae) collected in Mie Prefecture, Japan [115]. They characterized **30a** and **30b** by employing spectroscopic analyses including NOESY. The structures of **30a** and **30b** consist of polycyclic polypropionates that contain a δ-pyrone ring and a spiroketal moiety. Auripyrones A **30a** and B **30b** exhibited cytotoxicity against HeLa S_3_ cells with IC_50_ = 0.26 and 0.48 μ/mL, respectively. Tautomycin 1 (**31**) (Figure 7) was isolated from *Streptomyces spiroverticillatus* from a soil sample collected in Jiangsu Province, China [116]. The name of tautomycin was chosen because it exists in a tautomeric mixture in solution. Compound **31** exhibited strong antifungal activity against *Sclerotinia sclerotiorum,* and induced morphological change (blebbing) of the human erythroid leukemia cell K562. Compound **31** is also a potent inhibitor of the spread of human myeloid leukemia cell HL60 [116], and the protein phosphatases type 1 and type 2A [117]. The structure of polyether **31** consists of 13 chiral centers and was determined by chemical degradation and conformational calculations [118]. Other characteristic structural features of tautomycin 1 **31** are the tetramic acid derivative termini linked to an ester, internal and terminal ketones, and a polypropionate spiroketal moiety. Aplysiatoxin **32** (Figure 7) was first isolated and purified from the marine gastropod mollusc *Stylocheilus longicauda* and the structure of **32** was elucidated employing UV–Vis, NMR, IR, and MS data analysis and chemical degradation [119]. The absolute configuration of **32** was established using optical, NMR, X-ray crystallographic analysis, and an examination of the degradation products of **32** [120]. The macrolactone **32** has a tetrahydropyran–tetrahydropyran (THP–THP) spiro system and side chain with a bromo phenol moiety. Aplysiatoxin **32** and related metabolites have shown to be potent tumor promoters [121].

#### 1.2.2. Other Related Polypropionates

Bengamide E **33** (Figure 7) was first isolated from an undescribed Jaspidae sponge collected in the Benga lagoon of the Fiji Islands. The structure and stereochemistry including the chirality of the substituted ɛ-caprolactam ring of bengamide E **33** was established by comparing the structure of the analogs bengamides A and B through extensive spectroscopic studies and chemical degradation. Bengamide E **33** was also extracted and characterized from *Jaspis* cf. *coriacea* from Fiji [122] and *Pachastrissa* sp. from Djibouti [123]. Bengamide **33** has shown in vitro cytotoxic activity against the MDA-MB-435 (IC_50_ = 4–7 nM) human breast carcinoma cell line and also demonstrated an in vitro anthelminthic activity [122]. Multiplolide A **34** (Figure 7) is a ten-membered lactone (decalactone) that was isolated from the broth of *Xylaria multiplex* BCC 1111 [124]. In 2001, the structure of **34** was elucidated by employing spectroscopic and spectrometric analysis, but the relative configuration of the epoxide moiety could not be assigned. Then, in 2008, the absolute configuration was assigned after the total synthesis of **34** [125]. Multiplolide A **34** exhibited antifungal activity against *Candida albicans* (IC_50_ = 7 μg/mL).

## 2. Polypropionate Epoxide-Based Approaches

### 2.1. Kishi (Pioneer)

**Figure 8:**
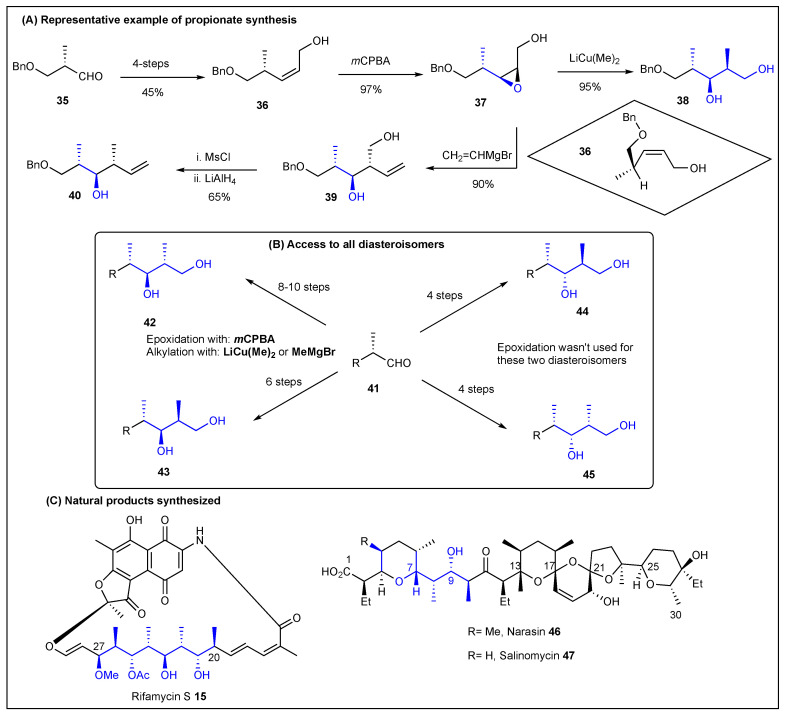
Kishi’s allylic epoxidation-based approach toward polypropionates.

It was 1979 when Kishi reported a stereo- and regioselective method for the synthesis of propionates from allylic alcohols [126,127]. The synthetic approach started from chiral aldehydes such as **35** to induce stereoselectivity, which after 4-steps was converted into chiral allylic alcohol **36** (Figure 8). Adduct **36** was then treated with *m*CPBA to form epoxide **37** in a 97% yield. This epoxide was then regioselectively opened to form propionate **38** using LiCu(Me)_2_ in a 95% yield. Grignard reagents such as (CH_2_=CHMgBr) can also be used to increase the propionate backbone to produce terminal olefins that can be further functionalized (Figure 8). In simple terms, Kishi’s approach to propionates relies on the use of *m*CPBA as the epoxidating agent [128] and either the Gilman or Grignard reagents for the alkylation step. It is important to note that Kishi was also able to synthesize all four diastereomers by either the pioneer epoxide approach or through hydroboration-oxidation conditions (Figure 8, middle) [126,127]. To validate the scope, Kishi and coworkers worked on the total synthesis of rifamycin S [129,130,131] and the partial synthesis of narasin and salinomycin [132] (Figure 8, bottom).

### 2.2. Katsuki

**Figure 9:**
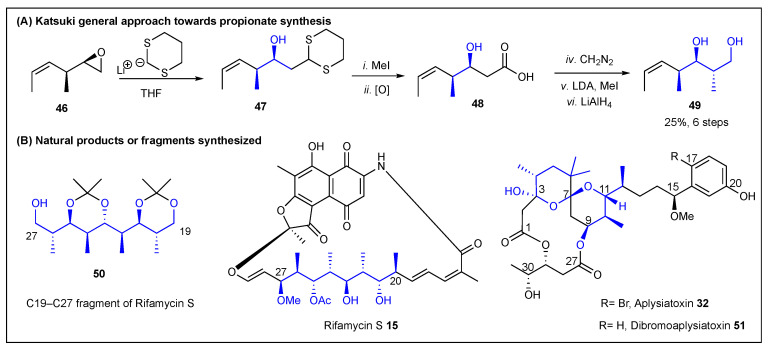
Katsuki’s dithianative epoxide-ring opening approach toward polypropionates.

A few years later (1989), Katsuki and coworkers reported a different approach toward polypropionates [133]. Katsuki’s approach is based on an epoxide ring-opening using 2-lithio-1,3-dithiane [134]. Figure 9 shows the synthetic sequence, which starts from a dithianative epoxide ring opening of **46** to produce dithioacetal **47**. This intermediate was treated with iodomethane to allow an alkylative desulfurization that released an aldehyde, which was then oxidized with NaClO_2_ to afford carboxylic acid **48**. Then, esterification with CH_2_N_2_, followed by α-methylation and a reduction with lithium aluminum hydride, produced the expected propionate **49**. Further elaboration of intermediate **49** produced the rifamycin S fragment **50**. It is worth noting that Katsuki utilized either VO(acac)_2_ or WO_5_·HMPA for epoxidations [133]. Furthermore, the same synthetic approach was used during a formal total synthesis of aplysiatoxin **32**; this methodology could also be used to synthesize dibromoaplysiatoxin (Figure 9, bottom right).

### 2.3. Marshall

**Figure 10:**
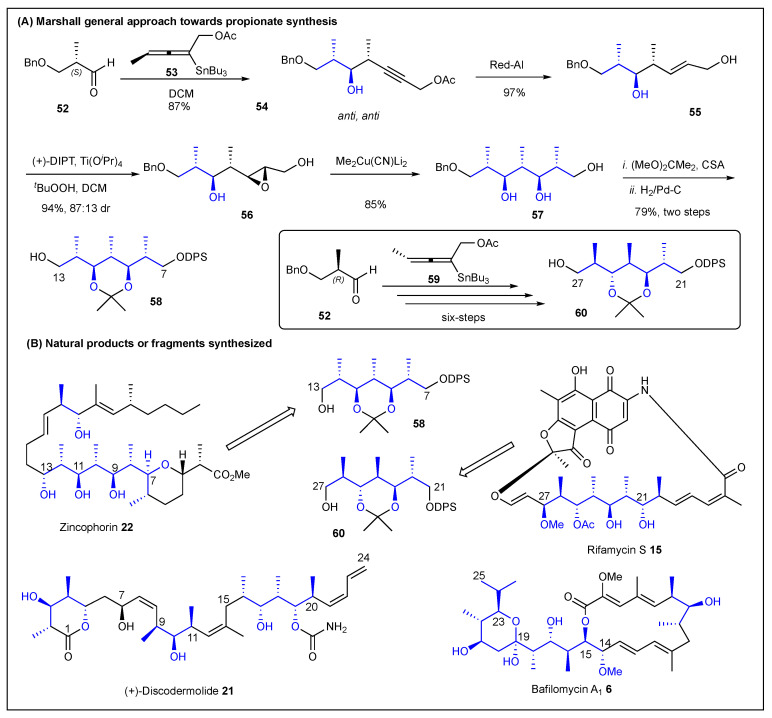
Marshall’s allenylstannane epoxide-based approach toward polypropionates.

In 1998, Marshall reported the addition of chiral allenylstannanes such as **53** to chiral aldehydes (e.g., **52**) to produce homopropargylic alcohols with two contiguous *anti-anti* stereocenters (Figure 10, top left) in an 87% yield [135]. The homopropargylic alcohol **54** was then reduced with Red-Al to allylic alcohol **55**. Then, using Sharpless asymmetric epoxidation conditions, the expected product **56** was observed with an 87:13 dr. The addition of Me_2_Cu(CN)Li_2_ opened the epoxide by regio-and stereoselective addition of a methyl group to afford polypropionate **57** in an 85% yield. Finally, the protection and deprotection sequences delivered zincophorin’s C7–C13 fragment (**58**). Likewise, this reiterative epoxidation-methyl cuprate sequence to transform allylic alcohols into polypropionates was used to prepare rifamycin-S’s C21–C27 fragment (**60**), albeit employing other enantiomers during the initial allenylstannane addition (Figure 10, middle) [135]. Due to the fact that by using chiral allenylstannanes, five carbons can be added to the backbone in a single step, so this polypropionate approach is efficient and useful for the synthesis of natural products. For instance, Marshall and coworkers reported the total synthesis of (+)-discodermolide with an excellent stereocontrol observed [136]. They also reported the stereoselective synthesis of bafilomycin A1′s C15–25 fragment, this time using allenylzinc as the key reagent (Figure 10, bottom) [137].

### 2.4. Miyashita

**Figure 11:**
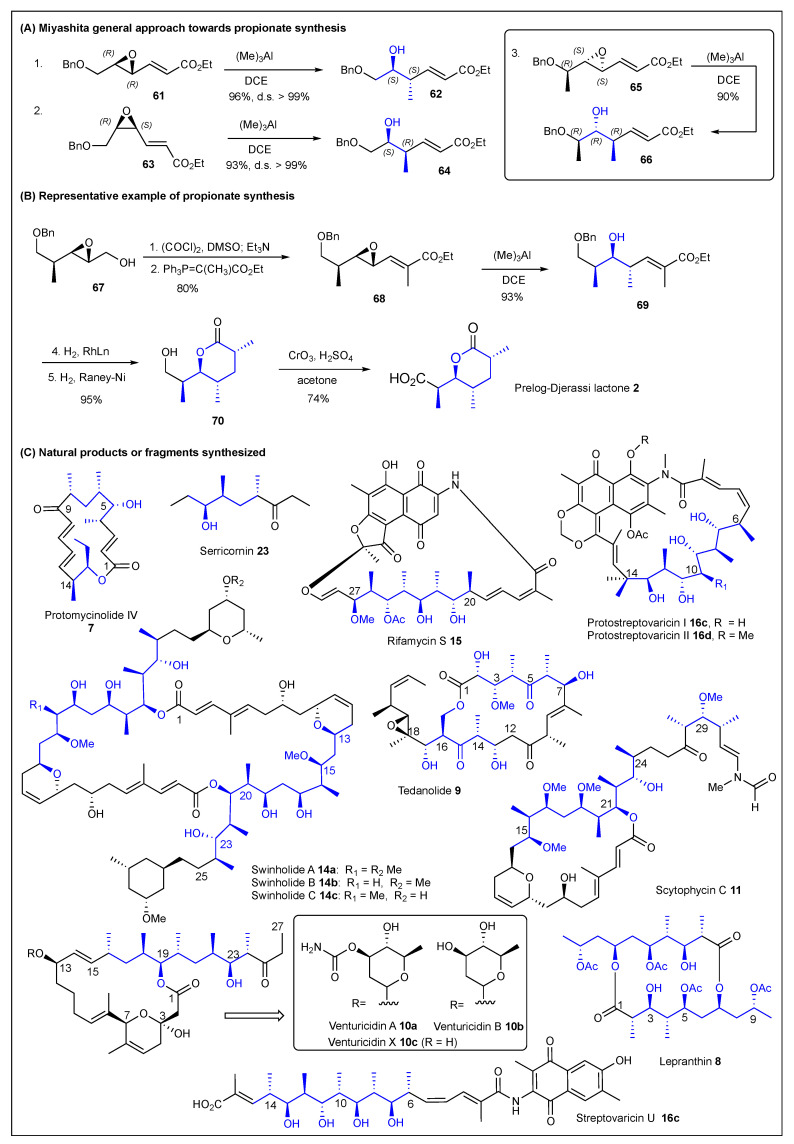
Miyashita’s trimethylaluminum epoxide-ring opening toward polypropionates.

Miyashita was one of the major contributors to publications related to the synthesis of fragments and natural products containing polypropionate subunits. His methodology relies on the use of trimethylaluminum for the regio- and stereospecific methylation of epoxides. In 1991, his group reported a new iterative method for the construction of polypropionate chains from epoxy acrylates [138]. The method takes advantage of optically active epoxy acrylates that can undergo stereospecific methylation with 10 equiv. of (Me)_3_Al to afford highly diastereoselective adducts. For example, the (*E*)-epoxy acylate **61** produced *anti* adduct **62**, while the (*Z*)-epoxy acylate **63** gave *syn* adduct **64** (Figure 11, top) in very high yields and diastereoselectivity. It is important to note that having adjacent chiral centers does not affect the yields or diastereoselectivity [138]. Using this stereospecific methylation approach, Miyashita and co-workers synthesized Prelog–Djerassi lactone **2** in six synthetic steps (Figure 11, middle) [139]. They also reported the formal synthesis of protomycinolide IV **7** [139]. In addition, several other fragments and/or natural products have been prepared by Miyashita et al. For instance, the following polypropionate-containing-natural products: (−)-serricornin **23** [140], rifamycins **15** [141], streptovaricin U **16c** [142], protostreptovaricin I and II (**16c-d**) [142], scytophycin C **11** [143,144,145], swinholides A-C **14a-c** [146], tedanolide **9** [147], lepranthin **8** [148], and venturicidins **10a-c** [149] were all prepared from epoxides and trimethylaluminum for the regio- and stereospecific methylation (Figure 11, bottom). It is worth noting that Miyashita also reported the use of *m*-chloroperoxybenzoic acid (*m*CPBA) for highly stereoselective epoxidations of chiral allylic alcohols [150] and organoselenium reagents for a chemoselective reduction of epoxides [151]; both important methodologies for the synthesis of polypropionates.

### 2.5. Prieto

**Figure 12:**
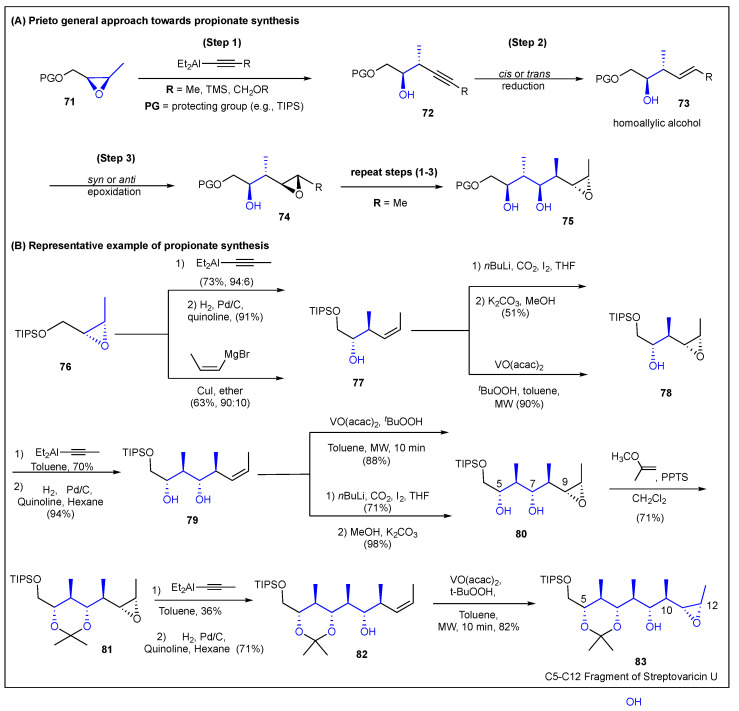
Prieto’s three step epoxide-based reiterative approach for polypropionate construction and the stereoselective synthesis of the C5–C12 fragment of streptovaricin U.

Prieto’s methodology is a relatively simple substrate-control approach and consists of three reiterative steps: (1) regioselective cleavage of epoxide **71** with an organoaluminium or organomagnesium reagents; (2) cis or trans reduction of the resulting alkyne **72**; (3) syn or anti epoxidation of the homoallylic alcohol **73** [152,153] to subsequently produce the 3,4-epoxyalcohol **74**. The application of the reiterative three step methodology produced a new propionate unit **75** (Figure 12). The absolute stereochemistry of the polypropionate fragment was introduced during the synthesis of the first epoxide for step one by employing the Sharpless asymmetric epoxidation [154]. The relative configuration of the methyl groups is defined by the cis/trans geometry of the epoxide precursor, which in turn is defined by the geometry of the alkene precursors. The configuration of the secondary hydroxy groups is defined by the configuration of the epoxide precursors. In 2009, Prieto et al. reported the stereoselective synthesis of the very challenging *all*-anti, the C6–C10 polypropionate chain **83** of streptovaricin U **16e** (Figure 12) [155]. For this, the syn-selective epoxidation of *cis*-homoallylic alcohols **77** and **79** were obtained by either applying the iodocarbonation/methanolysis procedure [40,156] or the VO(acac)_2_ catalyzed epoxidation reaction [153,157]. Then, the regioselective cleavage of epoxides **76**, **79**, and **81** with a propynyl aluminum reagent followed by a *cis* reduction produced homoallylic alcohols **77**, **79**, and **82** in good yields. Furthermore, the incorporation of the copper-catalyzed Grignard epoxide cleavage reaction [158] for the cleaving of epoxide **76** to complement the alkynyl alane approach [25,25,159,160] was also developed.

**Figure 13:**
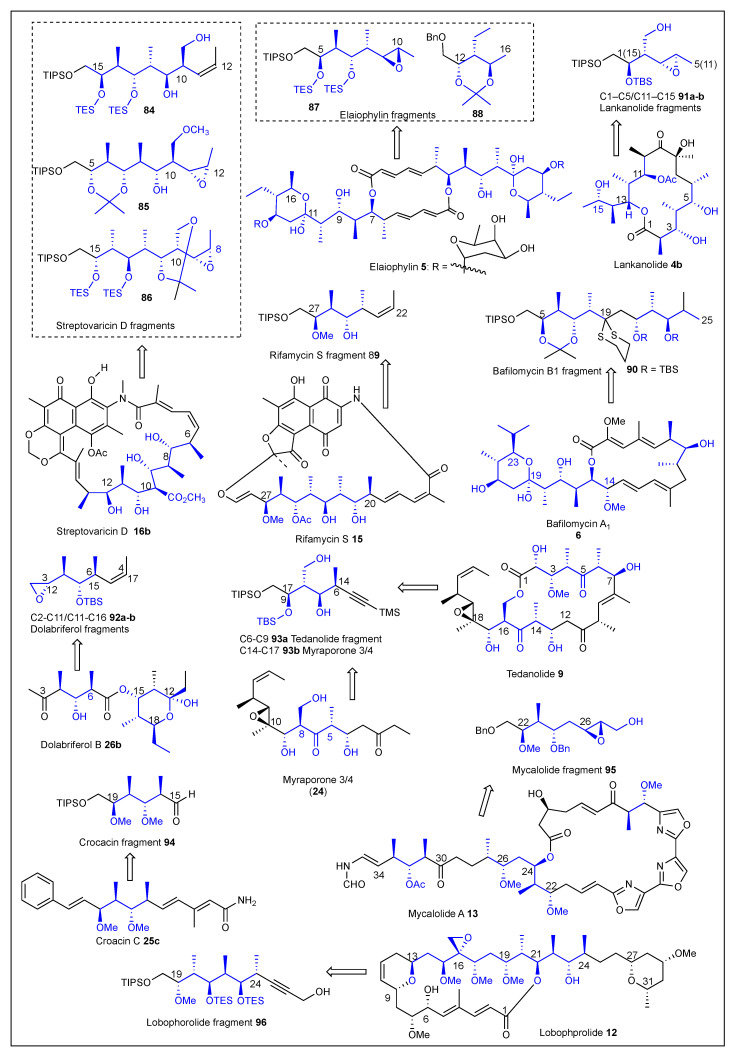
Polypropioante fragments and natural products reported by Prieto’s group.

To demonstrate the synthetic usefulness of this non-aldol approach for the elaboration of polypropionate-containing natural products, they applied a linear three-reaction sequence to the stereoselective construction of the stereohexads corresponding to the C15–C10 **84** [158], C5–C12 **85** [161], and C15–C8 **86** [161] polypropionate chains of streptovaricin D **16b** including the C10 carbomethoxy functionality needed for the elaboration (Figure 13). Prieto also reported the C5–C10 **87** [160] and triad C12–C16 **88 [161]** fragments of elaiophylin **5**, in addition to the stereotetrad C22–C27 **89** [158] fragment of rifamycin S **15** (Figure 13). In 2012, the linear, convergent, and enantioselective synthesis of the C14–C25 **90** fragment of bafilomycin A_1_ **6** was reported in a 16% overall yield. The process consisted of eight steps in its longest linear sequence (Figure **13)** using a dithiane substitution reaction to the coupling of the corresponding linear fragments [162]. This group has reported the convergent synthesis of optically active and common precursors of the C1–C5/C15–C11 segment **91a,b** of lankanolide **4b** [157] (Figure 13) and the convergent precursor for the C3–C9/C12–C18 fragments **92a,b** of dolabriferol B **26b** (Figure 13) [161]. They also reported a second-generation methodology in 2014 to introduce the hydroxymethyl moiety found at the C16 of tedanolide **9** and C18 of myraporone 3/4 (**24**) [159]. Specifically, they constructed an optically active stereotetrad **92** that is a common intermediate for the elaboration of C6–C9 **93a** and C14–C7 **93b** aliphatic chains of tedanolide **9** and myraporone 3/4 (**24**), respectively (Figure 13). The most recent paper reported by Prieto et al. describes the enantioselective and stereoselective synthesis of several polypropionate chains, which include the C15–C20 segment **94** of crocacin **25** and the polypropionate chains of C22–C27 **95**, mycalolide A **13**, and C19–C24 **95** of the lobophorolide **12** natural products (Figure 13) [161]. In summary, this reiterative, linear, and convergent methodology has demonstrated the versatility to synthesize stereotriads, stereotretrads, and stereohexads with regio-, enantio-, and diastereoselectivity in polypropionate natural products.

### 2.6. Sarabia

**Figure 14:**
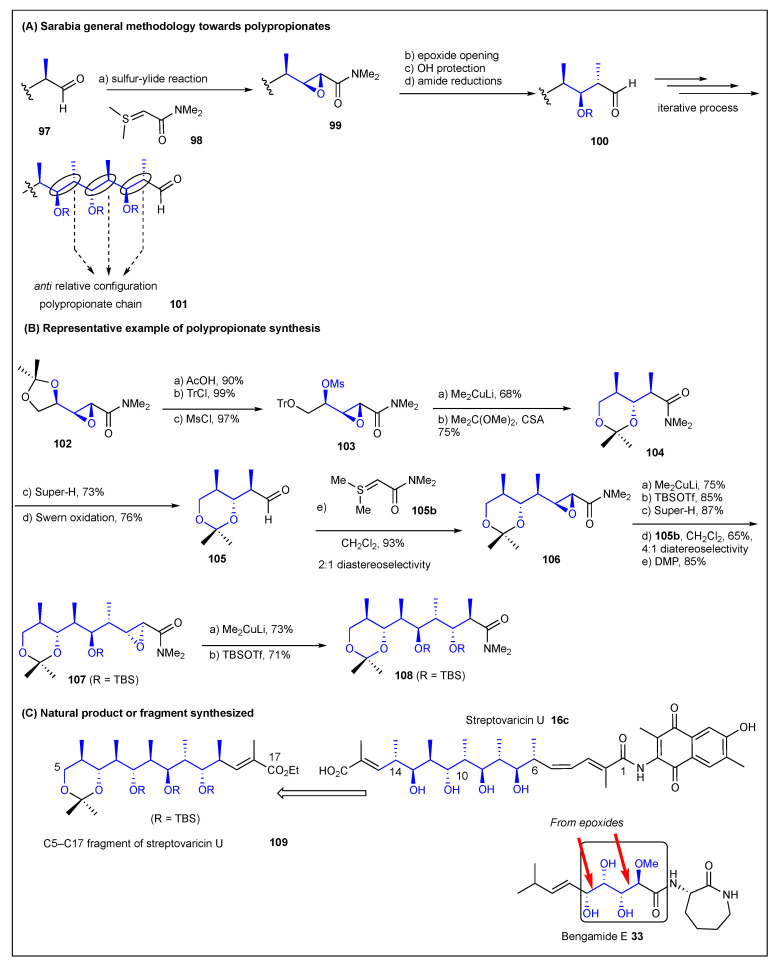
Sarabia’s methodology for the synthesis of polypropionates.

More recently, the group of Sarabia reported the stereoselective synthesis of polypropionate-type frameworks utilizing an epoxy amide-based strategy (Figure 14) [163]. The reiterative methodology of Sarabia consists of four consecutive steps: sulfur ylide reaction, oxirane opening, protection, and reduction of amide. The starting material is a chiral aldehyde **97** that is reacted with a sulfur ylide **98** in a stereoselective fashion induced by the presence of an asymmetric center at the α-position of aldehyde **97** (Figure 14). The resulting *trans* epoxyamide **99** is then subjected to the epoxide opening reaction mediated by a methyl organocuprate reagent, followed by the protection of the resulting alcohol as a silyl ether and the reduction of the amide to the alcohol. At this point, the oxidation of the alcohol provided a new aldehyde **100**, which was ready for the application of a second reaction sequence. This sequence provided the *anti*-relative configuration between the chiral centers present in the resulting polypropionate chain **101** (Figure 14). A collection of different chiral aldehydes was subjected to reaction with sulfur ylide. The majority showed remarkable stereofacial differentiation, providing a major diastereomer, in contrast to other aldehydes that displayed poor to no stereoselectivity. Despite the difficulties encountered for the preparation of some epoxide amides with respect to the diastereomeric yields, Sarabia was able to prepare various stereo-*n*-ads such as **108** including the synthesis of the C5–C17 polypropionate chain of streptovaricin U **109** (Figure 14) [164]. In 2010, Sarabia and co-workers reported on the stereoselective preparation of bengamide E **33** and analogs using chiral sulfur ylides [165]. Specifically, they achieved the synthesis of the tetraol polyketide fragment present in bengamide E **33** by using two *trans***-**epoxides synthesized by employing Sharpless asymmetric epoxidation (SAE). This was then followed by conversion into epoxyamides to apply the oxirane-ring-opening process with alkylborates, catalyzed by palladium (0) to yield the corresponding ring opened *syn*-diol products (Figure 14) [166].

### 2.7. Jung

**Figure 15:**
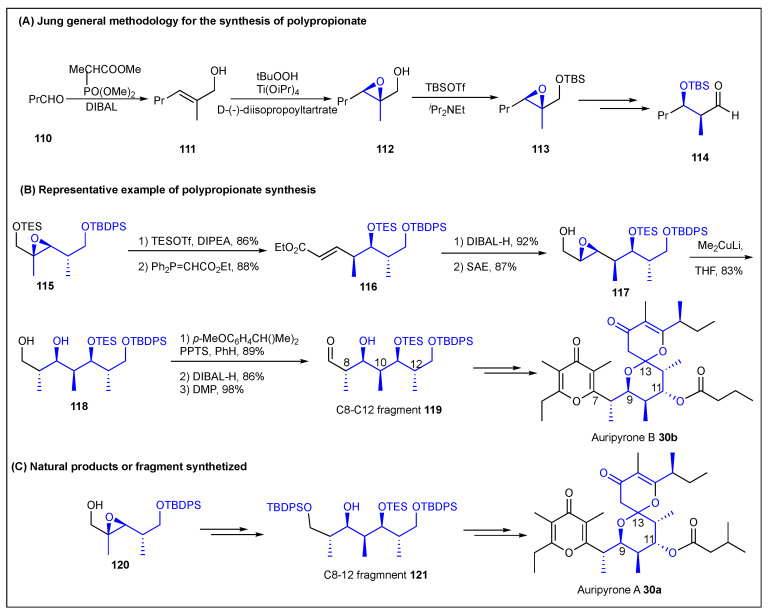
Jung methodology for the synthesis of polypropionate.

Jung et al. developed a “non-aldol aldol” protocol for the synthesis of polypropionates that involves the stereoselective olefination of the aldehyde **110**, followed by a hydride reduction to produce the allylic alcohol **111**. Sharpless asymmetric epoxidation of **111** yielded epoxy alcohol **112** (Figure 15) [24,167,168]. Silylation of the primary alcohol in **112** and the final Lewis acid catalyzed the rearrangement of the epoxy silyl ether **113**, generating the desired *syn* or *anti* propionate unit product **114**. The epoxy silyl ether epoxide **112** is activated with the Lewis acid TBSOTf, and an intramolecular hydride transfer cleaves the epoxide **113**, generating an optically active aldehyde **114** (Figure 15). *E*-allylic alcohols like **111** generate *syn* adducts whereas *Z-*allylic epoxides generate *anti* adducts. In 1999, Jung et al. examined the Lewis acid promoted rearrangements of several allylic epoxides and their derivatives as a method for the preparation of compounds that can be used in the synthesis of natural products such as Tedanolides [167]. Jung et al. first reported the total synthesis of auripyrone B **30a** from the known epoxide **120** in 18 steps and a 17% overall yield. They employed a non-aldol Lewis acid catalyzed rearrangement of an epoxy silyl ether strategy combined with a Paterson aldol process (Figure 15) [168]. In 2010, Jung et al. also reported the total synthesis of auripyrone B **30b** in only 20 steps in an 8% yield starting from optically active epoxy silyl ether **115** using a syn non-aldol aldol/*anti* cuprate opening strategy to produce the C8–C12 polypropionate fragment **119** (Figure 15) [169].

### 2.8. McDonald

**Figure 16:**
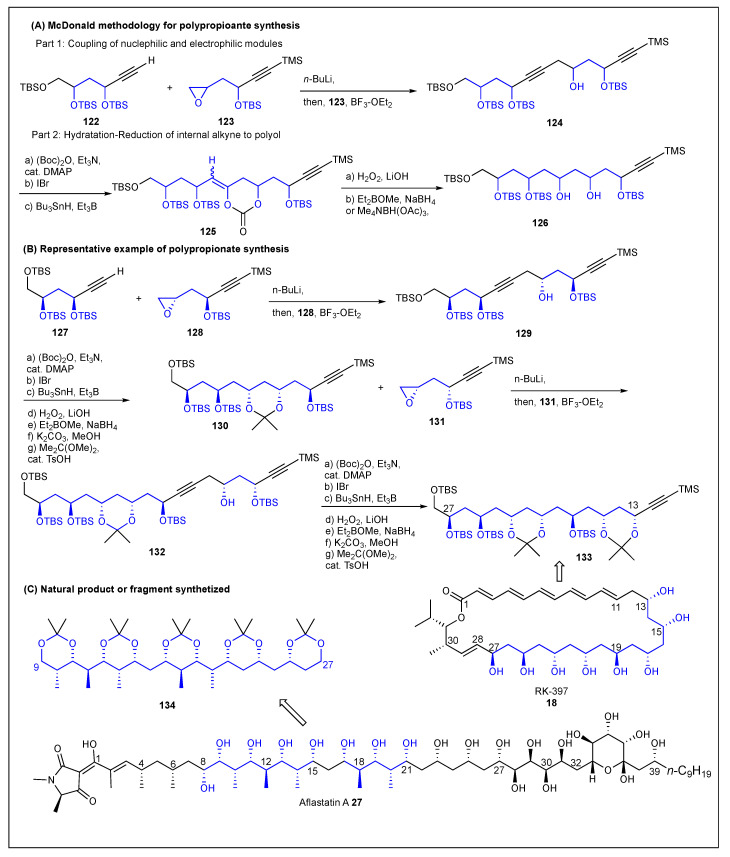
McDonald epoxide-ring opening toward polypropionates.

In 2002, McDonald and co-workers reported a new strategy for the construction of alternating 1,3-polyols based on two parts. The first, cross-coupling of six-carbon alkynol **122** (nucleophile) and the six-carbon epoxyalkynol **123** (electrophile) modules by epoxide opening. Second, the resulting internal alkyne **124** was hydrated to form **125** and then reduced with either 1,3-*anti*-15 or *syn*-stereoinduction to form the polyol fragment **126** (Figure 16) [170]. They reported the preparation of the C13–C27 polyol fragment **134** of the macrolide polyne RK-397 **18**. The cross-coupling between terminal alkyne **127** with epoxide **128** produced the dialkyne **129**. The hydration-reduction of the resulting internal alkyne in **129** produced the polyol fragment **130**, which was then coupled with epoxide **131**. The synthesis concluded with regio- and stereoselective synthesis of the polyol fragment **133** (Figure 16) [170]. In another paper, McDonald and co-workers reported the synthesis of the C9–C27 polypropionate fragment **134,** which is the degradation product of aflastatin A **27** using the alkyne-epoxide cross-coupling strategy [171]. Particularly, the polypropionate fragments **134** and **135** were obtained by employing the stereoselective vanadium catalyzed and iodocarbonation/methanolysis epoxidations, respectively.

### 2.9. Lipshutz

**Figure 17:**
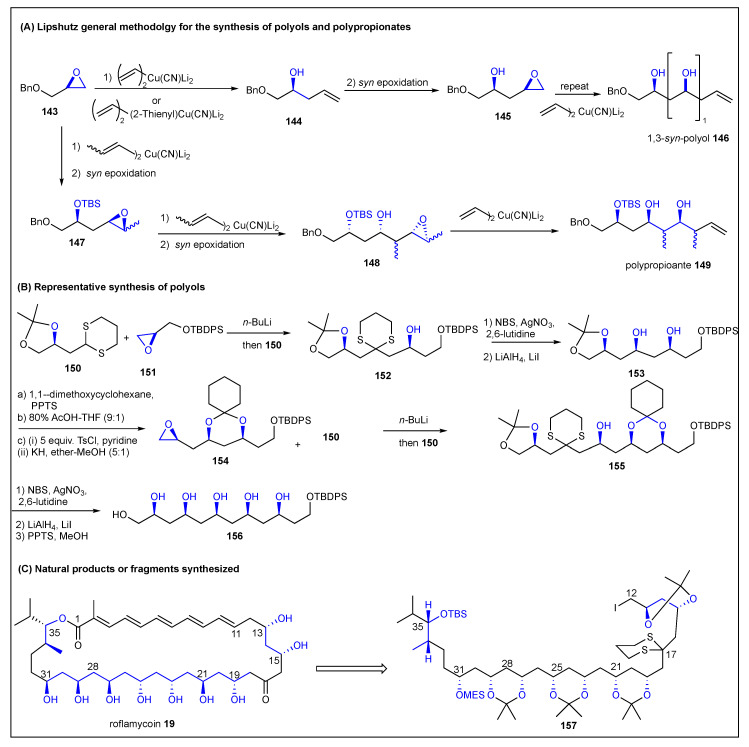
Lipshutz methodology for the synthesis of polypropionate.

Lipshutz and co-workers, in 1984, described a two-step methodology for the formation of all *syn*-1,3-polyols **146.** They employed the vinyl-lithium-based higher order cyano-cuprates to cleave the monosubstituted epoxide **143** and **145**, followed by epoxidation of the resulting homoallylic alkenol **144** (Figure 17) [172,173]. An alternative methodology for the synthesis of polypropionate chains was also reported by Lipshutz and Barton in 1988 starting with the regiospecific cleavage of epoxide **143** with the *cis*- or *trans*-propenyl high order cuprates (Figure 17) [174]. The cleavage of the disubstituted 3,4-epoxy alcohol **147** yielded the desired polypropionate products **148** and **149** in variable regioselectivities. In contrast, the epoxide cleavage with the vinyl cuprate reagent enhanced the regioselectivity at latter stages, providing 1,3-diol exclusively. In this sequence, the epoxidation of the homoallylic alcohols was achieved in high stereoselectivities using the iodocarbonation/methanolysis sequence, while the VO(acac)_2_ protocol was also quite efficient. In 1988, Lipshutz and co-workers also reported on the construction of the desired all *syn*-polyol array **153** and **156** by employing the coupling reaction between dithiane **150** with epoxides **151** and **154** to obtain the products **152** and **155**, respectively. Hydrolysis of dithianes followed by a highly *syn-*selective LiAlH_4_-LiI reduction method produced all of the *syn*-polyols **153** and **155** (Figure 17). In 1989, this epoxide and dithiane coupling strategy was applied to the construction in an optically pure form of the C12–C35 fragment **157** of the 36-membered polyene macrolide roflamycoin **19**. Unfortunately, the stereochemistry of the eleven chiral centers in this year was unknown and they assumed that **19** had all of its polyhydroxyl groups in a *syn*-relationship (Figure 17) [175].

### 2.10. Nicolaou

**Figure 18:**
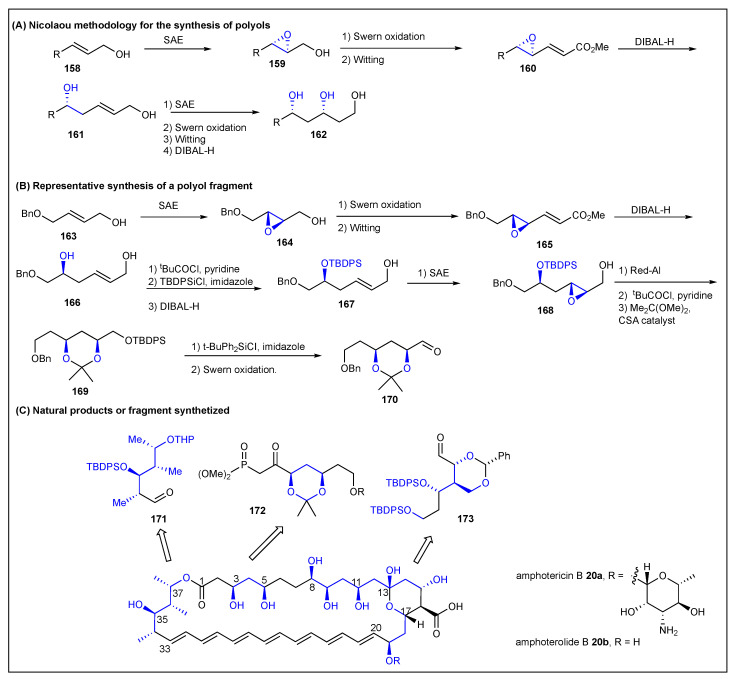
Nicolaou’s methodology for the synthesis of polyols.

Nicolao and Uenishi, in 1982, reported an approach that utilizes the allylic alcohol **158** as a starting material, converted in epoxyalcohol **159** by the SAE reaction. This was then converted into the γ,δ-epoxy-α,β-unsaturated ester **160** before a regioselective hydride reduction with DIBAL to afford the allylic and homoallylic alcohol **161** (Figure 18) [176]. The application of the aforementioned sequence generated the 1,3-*syn*-polyol fragment **162**. Years later, Nicolaou and co-workers reported the synthesis of various key building blocks **170** to **173** toward the total synthesis of amphotericin B **20a** and amphoterolide B **20b** (Figure 18) [177]. They envisaged a convergent synthesis to construct polyol fragments **170** and **172**. To achieve this, **163** was converted into epoxide **164** by the SAE reaction, then Swern oxidation followed by the Witting reaction, which produced the conjugated epoxy ester **165**. A protection–deprotection strategy provided **167**, which was then converted into the epoxide **168**. The hydride reduction of **168** provided the *syn*-1,3-diol unit **169**, which is the common precursor for the divergent synthesis of **170** and **172** polyol fragments (Figure 18). For the synthesis of the fragment **172** present, Nicolaou employed an epoxide cleavage reaction with two organometallic reagents; Et_2_AlC≡CCH_2_OTBDPS and CH_2_=CHMgBr catalyzed by copper, and SAE as the key step for its elaboration. The total synthesis of amphotericin B **20a** and amphoterolide B **20b** was achieved by the efficient coupling of **170**, **171**, **172**, and **173** fragments by employing four aldehyde-phosphonate type condensation reactions and an esterification reaction [178].

### 2.11. Polypropionate Synthesis by Others

**Figure 19:**
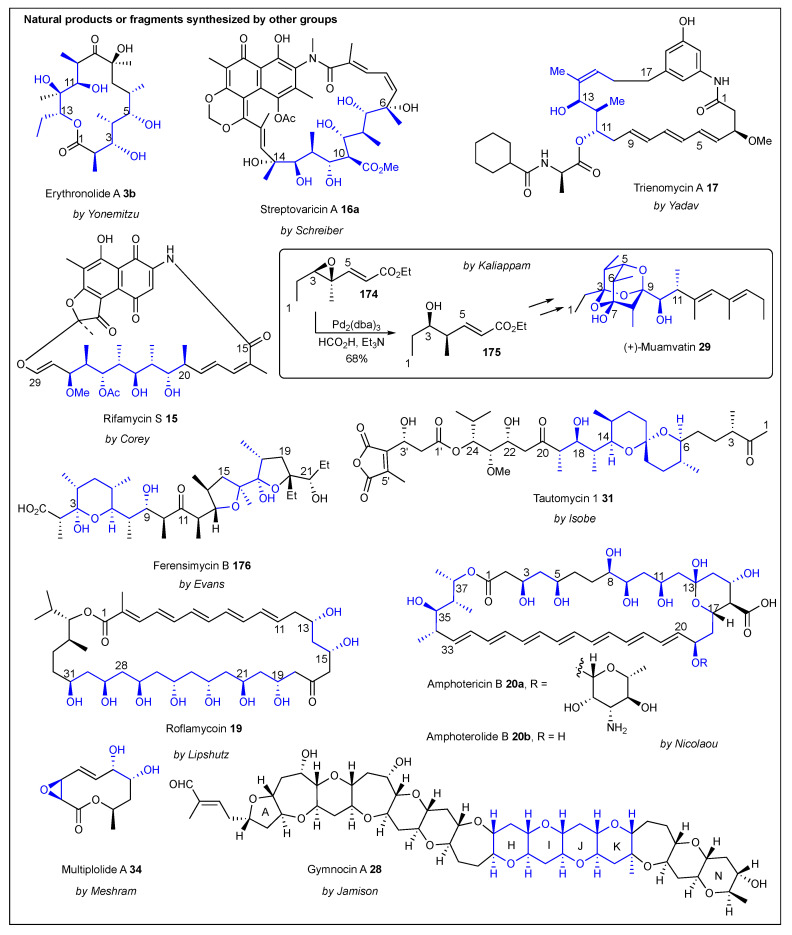
Natural products synthesized following the epoxide-based approaches (several authors).

Aside from the synthetic approach toward polypropionates by the research groups described above, there are other research groups that have contributed to this field with one or two publications (also see Table 1). Figure 19 depicts several natural products that these research groups have targeted, with reports varying from the synthesis of fragments to total syntheses. With few exceptions, these other methods are analogs to the synthetic approaches described above, since they follow slightly modified protocols and somewhat different starting materials. However, they do complement the epoxide-approach toolkit. In 1979, Corey reported the use of an epoxide intermediate to construct the C15–C29 chain of rifamycin **15** (Figure 19) [179]. However, it was not until 1982 when he disclosed that a vinyl cuprate was used to open the epoxide [180]. A year earlier (1981), Lipshutz documented the use of high order mixed organocuprates in substitution reactions at unactivated secondary centers [181,182]. In 1982, Lipshutz also documented their reactivity toward epoxides [183], followed by their application in other organic reactions [176,184,185] and the synthesis of fragments of polypropionates [175]. In 1989, Yonemitsu et al. described an alternative synthesis toward two fragments C1–C5/C9–C15 of erythronolide A **3b** via regio- and stereoselective reductive ring opening. The key of their methodology was to utilize trisubstituted epoxides (the methyl group pre-installed) and B_2_H_6_ as a reducing agent [186]. Schreiber also reported stereochemical studies of epoxides toward the ansa chain of streptovaricin A **16a**. He also took advantage of trisubstituted epoxides but used an intramolecular ring opening facilitated by a nearby urethane that was deprotected with Et_2_AlCl [187]. In 1993, Yadav et al. also utilized trimethylaluminum, an epoxy acrylate intermediate to synthesize fragment C9–C16 of trienoycin A **17** [188]. In addition, Zhai used Me_2_CuLi to open chiral allylic epoxides [189], Haddad used organocuprates and organoaluminium reagents [190], Sato used NaBH_4_ to open trisubstituted trimethylsilyl epoxides [191], Mulzer used high order organo cyanocuprates to open two consecutive epoxides [192], and Tan used SmI_2_ to reduce epoxides and MeMgCl or Me_3_Al to ring-open the epoxide while installing the methyl group [193]. More recently (2020), Kaliappan employed palladium mediated stereoselective reduction (ring opening) of the trisubstituted epoxide **174** to produce propionate **175** in a 68% yield [194]. It is important to note that this is the first example of palladium chemistry used to construct polypropionates, which led to the synthesis of the C1–C10 fragment of muamvatin **29** (Figure 19, middle).

## 3. Other Epoxide Based-Syntheses of

### Polyols and Polyethers

It is well-known that all of the methodologies described above are paired with the aldol chemistry developed by Evans and others. Nonetheless, Evans also reported the use of epoxides for the synthesis of polyethers such as ferensimycin B **176** [199]. In this case, a neighboring hydroxy group performed an intramolecular epoxide ring opening to form an ether, which in turn could lead to polyethers after a few iterations. Lipshutz also contributed with his high order cuprate protocol for the synthesis of polyols [200] such as roflamycoin **19** [174,176] (Figure 17 and Figure 19). Isobe also utilized Grignard or Gilman reagents to regioselective open epoxides to either form polypropionates or polyethers such as tautomycin 1 **31** [197] (Figure 19). DIBAL and REDAL were the reducing agents of choice by Nicolaou for the regioselective reduction of chiral disubstituted epoxides [177] toward polyols such as amphoterecin B **20a** and amphotheronolide B **20b** [178] (Figure 18 and Figure 19). The total synthesis of multiplolide A **34** was reported by Meshram et al. Treating a disubstituted epoxide with Sc(OTf)_3_ and water afforded the expected diol in good yield [196]. Aside from the well-known Sharpless dihydroxylation reaction to make polyols, he also used Red-Al to open epoxides in his search for synthetic alternatives toward polyols [201]. Furthermore, Jamison reacted a polyepoxide with water at 60 °C for days to produce a polyether intermediate used to construct the HIJK rings of gymnocin A **28** [198] (Figure 19, bottom) and Table 1.

## 4. Conclusions

This manuscript describes the isolation and characterization of approximately 30 polypropionate and related natural products that have shown antimicrobial activities against bacteria, fungi, and yeast. Some of the polypropionates also displayed cytotoxicity against different types of cancer lines. The first epoxide-based methodology for polypropionate synthesis was reported by Kishi and collaborators in 1980 and was applied to the first synthesis of the ansa chain of rifamycin S **15**. Kishi’s five step methodology was based on Sharpless asymmetric epoxidation followed by lithium dimethyl cuprate ring opening to construct the propionate units. Yamagishi et al. reported the synthesis of two types of Kishi’s intermediates, one for the construction of the *ansa* chain of rifamycin S **15** and the other for the formal synthesis of the aplysiatoxin **32** C1–C21 fragment, based on the stereoselective vanadium-catalyzed or tungsten-based epoxidations of *cis*-homoallylic alcohols and the asymmetric epoxidation of allylic alcohols. Marshall et al. were also influenced by the pioneering work of Kishi and employed a Lewis acid mediated allenylmetal addition to chiral aldehydes followed by the Kishi reiterative asymmetric epoxidation-methyl cuprate sequence. The research groups of Miyashita (1992 to 2011) and Prieto (2004 to 2017) applied their epoxide-alkynylaluminum cleavage approaches to the formal and total synthesis of several polypropionate natural products in common. Miyashita’s group employed the regiospecific cleavage of γ,δ-epoxy acrylates with trimethylaluminum as the key steps while Prieto’s reiterative methodology reported the stereoselective vanadium-catalyzed epoxidation of homoallylic alcohols, followed by the epoxide-ring opening with alkynylaluminum reagents or alkenyl Grignards as the crucial steps for the elaboration of propionate motifs. Remarkably, McDonald’s group developed a cross-coupling with elaborated alkynyllithium and epoxyalkyne fragments to construct long polypropionate chains with good regioselectivity. Jaminson was the only group that employed SAE for the construction of *trans*-syn-fused polycyclic ethers by an epoxide-opening cascade reaction promoted by water. The groups of Nicolaou and Yonemitzu based their epoxide methodology on the regioselective hydride ring-opening trisubstituted 2,3-epoxyalcohols for the construction of the 1,3-polyols and polypropionate fragments, respectively. Lipshutz and Nicolaou based their epoxide-cleavage methodologies to the stereoselective synthesis of 1,3-diols motifs found in polyene macrolides. The most recent report was made in 2020 by Kaliappan et al., which reported the synthesis of the polypropionate fragment of Muamvatin using palladium chemistry to open their epoxide. The most successful organometallic reagents to introduce methyl groups by the cleavage of epoxides were Me_3_Al and Me_2_CuLi. Indeed, the Kishi, Marshall, Sarabia, Jung, and Isobe groups employed a methyl cuprate reagent, while Miyashita and Yadav selected the methyl aluminum reagent to construct the polypropionate fragments. Particularly, the Yamagishi, Prieto, Isobe, and Lipshutz groups reported the cleavage of hindered 3,4-epoxy alcohols with dithianes for the convergent synthesis of long polypropionate and polyol chains.

It is important to note that most of the reviewed synthetic approaches were developed/reported a few decades ago, with a clear decline in the number of publications in the past decade. However, it is believed that this review should help to put these methods back into the limelight. Thus, an incremental interest on the topic is expected in the near future, particularly for methods utilizing transition metals or mild reaction conditions.

In brief, this review condenses the known reports of epoxide-based approaches toward polypropionates, which are attractive structural motifs in synthetic organic chemistry and the total synthesis of natural products. As described above, the presence of propionate moieties in natural products has shown good antimicrobial activity. Therefore, it is believed that the known literature and this review will be beneficial in the design of novel approaches and the synthesis of other important natural products in the near future.

## Figures and Tables

**Table 1:** Summary of the formal and total syntheses of polypropionates and related natural products by research group (chronological order).

Group	Year	Fragment	Natural Product	Ref.
Kishi	1980	C29–C15	Rifamycin S **15**	[129]
	1980	Total synthesis	Rifamycin S **15**	[130]
	1981	C29–C15 2nd generation	Rifamycin S **15**	[131]
	1987	Left half	Narasin	[132]
Katsuki	1989	C19–C27 (Kishi’s intermediate)	Rifamycin S **15**	[133]
	1991	C1–C21 (Kishi’s intermediate)	Aplysiatoxin **32**	[134]
Marshall	1998	C7–C13	Zincophorin **22**	[135]
		C21–C27	Rifamycin S **15**	
	1998	Total synthesis	(+)-Discodermolide **21**	[136]
	2000	C15–C25	Bafilomycin A_1_ **6**	[137]
Miyashita	1992	Total synthesis	Prelog-Djerassi lactone **2**	[139]
		Formal synthesis	Protomycinolide IV **7**	
	1993	Total synthesis	(−)-Serricornin **23**	[140]
	1993	C27–C20	Rifamycins **15**	[141]
	1996	C17–C5	Streptovaricin U **16e**	[142]
		C17–C5	Protostreptovaricin I & II (**16c-d**)	
	1998	C19–C32	Scytophycin C **11**	[143]
	1999	C13–C25	Swinholides A-C **14a-c**	[146]
	2003	C1–C18	Scytophycin C **11**	[144]
	2003	Total synthesis	Scytophycin C **11**	[145]
	2005	C13–C23	Tedanolide **9**	[147]
	2011	C1–C10	Lepranthin **8**	[148]
	2011	C15–C17	Venturicidins **10**	[149]
Prieto	2004	C5–C10	Streptovaricin D **16b**	[153]
		C5–C12	Streptovaricin U **16e**	
	2005	C15–C10	Streptovaricin D **16b**	[158]
		C24–C27	Rifamycin S **15**	
	2007	C5–C10	Elaiophylin **5**	[160]
	2009	C6–C10	Streptovaricin U **16e**	[155]
	2011	C1–C4/C12–C15	Lankanolide **4b**	[157]
	2012	C14–C25	Bafilomycin A_1_ **6**	[162]
	2014	C14–C17	Tedanolide **9**	[159]
		C6–C9	Myraporone ¾ (**24**)	
	2017	C20–C15	Crocacins **24a-d**	[161]
		C3–C9/C12–C18	Dolabriferol B **26b**	
		C34–C22 and C22–C27	Mycalolide A **13**	
		C19–C24	Lobophorolide **12**	
		C5–C12 and C15–C8	Streptovaricin D **16b**	
		C12–C16	Elaiophylin **5**	
Sarabia	2007	C1–C13	Streptovaricin U **16e**	[164]
	2010 and 2013	Total synthesis	Bengamide E **33**	[165,166]
Jung	2010	Total synthesis	Auripyrone B **30b**	[169]
McDonald	2004	C28–C11	RK-397 (**18**)	[195]
	2008	C9–C27	Aflastatin A **27**	[171]
Corey	1979	C15–C29	Rifamycin S **15**	[179]
Yonemitzu	1989	C9–C15 and C1–C5	Erythronolide A **3b**	[186]
Lipshutz	1989	C12–C35	Roflamycoin **19**	[176]
Schreiber	1990	C5–C15	Streptovaricin A **16a**	[187]
Evans	1991	Total synthesis	Ferensimycin B **176**	[196]
Yadav	1993	C9–C16	Trienomycin **17**	[191]
Isobe	1997	C26–C27	Tautomycin 1 (**31**)	[197]
Nicolaou	1998	Fragments **170**, **171**, **172**, and **173**	Amphoterolide B **20b** & Amphotericin B **20a**	[177]
	1998	Total synthesis	Amphoterolide B **20b** & Amphotericin B **20a**	[178]
Jamison	2009	HIJK rings	Gymnocin A **28**	[198]
Meshram	2013	Total synthesis	Multiplolide A **34**	[196]
Kaliappan	2020	C1–C10	Muamvatin **29**	[194]

## Data Availability

The data are contained within the article.
